# Mapping the refractive index with single plasmonic nanoantenna

**DOI:** 10.1038/s41598-018-21395-w

**Published:** 2018-03-01

**Authors:** S. Gurbatov, O. Vitrik, Yu. Kulchin, A. Kuchmizhak

**Affiliations:** 1 School of Natural Sciences, Far Eastern Federal University, Vladivostok, Russia; 2 Institute of Automation and Control Processes, Far Eastern Branch, Russian Academy of Science, Vladivostok, 690041 Russia

## Abstract

As the size of the state-of-the-art optical devices shrinks to nanoscale, the need for tools allowing mapping the local optical properties at deep sub-diffraction resolution increases. Here we demonstrate successful mapping the variations of the refractive index of a smooth dielectric surface by detecting spectral response of a single spherical-shape Ag nanoparticle optically aligned with a supporting optical fiber axicon microlens. We propose and examine various excitation schemes of the plasmonic nanoantenna to provide efficient interaction of its dipolar and quadrupolar modes with the underlying sample surface and to optimize the mapping resolution and sensitivity. Moreover, we demonstrate an lithography-free approach for fabrication of the scanning probe combining the high-quality fiber microaxicon with the Ag spherical nanoparticle atop. Supporting finite-difference time-domain calculations are undertaken to tailor the interaction of the plasmonic nanoantenna and the underlying dielectric substrate upon various excitation conditions demonstrating good agreement with our experimental findings and explaining the obtained results.

## Introduction

As functionality of advanced optical materials and corresponding devices becomes more dependent on their local properties, importance of an optical probing of the matter at a true nanoscale spatial resolution increases. The development of such superresolution techniques for precise nanoscopy of the local optical properties (for example, reflectivity or refractive index (RI)) of the various samples paves the way toward novel practical applications in integrated optics for the characterization of various nanophotonic devices recorded in the photosensitive materials, in microbiology - for marker-free study of the biological samples, etc^1–5^. Strongly localized and enhanced optical fields produced by a nanoscale optical antennas (resonant plasmonic nanostructures^6–10^ or non-resonant nanotips^11–13^) and single quantum emitters (fluorescent molecules^14^, nitrogen vacancy centers^15^ and lanthanide-based emitters) are usually used to probe the local optical properties of the sample surface at diffraction-unlimited resolution^16,17^. Interaction of such nanophotonic structures with the underlying substrate manifests as a perturbation of the scattering (emission) intensity, in particular, via coupling of the field confined near the nanoantenna to the far-field radiation. This signal is typically used to characterize and map the local optical properties of the sample surface or even hidden subsurface features^18,19^. Meanwhile, in the case of the smooth optically transparent samples, the scattered/emission intensity weakly reacts on the small RI deviations of the surface yielding in a poor contrast and lateral resolution^20^. Moreover, while the extensive efforts were undertaken to tailor the optical nanoantenna design to achieve ultimate mapping characteristics, fabrication of the scanning probe containing complex-shape resonant optical nanoantenna represents a technologically complex procedure requiring several expensive and time-consuming steps to be involved^7,8,21^ and increasing the cost of the probe, in its turn.

In this paper we demonstrate successful RI mapping of a smooth dielectric substrate by detecting “pure” spectral response of a single spherical-shape Ag nanoparticle attached and optically aligned with an optical fiber microaxicon (FMA) lens. The later is used as a rigorous support for the resonant nanoantenna as well as a lens collecting the characteristic scattering signal. We propose and examine various excitation schemes of the plasmonic nanoantenna to provide efficient interaction of its dipolar (DP) and quadrupolar (QD) modes with the underlying sample surface and to optimize the mapping resolution and sensitivity. Namely, we show that by taking advantages of the strong energy localization of the QD mode and high sensitivity to the local dielectric environment of the DP one, it is possible to achieve superior lateral resolution in mapping of the local optical properties combined with the reasonably high sensitivity. Moreover, we suggest an easy-to-implement lithography-free protocol allowing fabrication of the scanning probe combining the high-quality fiber microaxicon and the Ag spherical nanoparticle atop.

## Experimental section

### Fabrication of the probe

**Figure 1:**
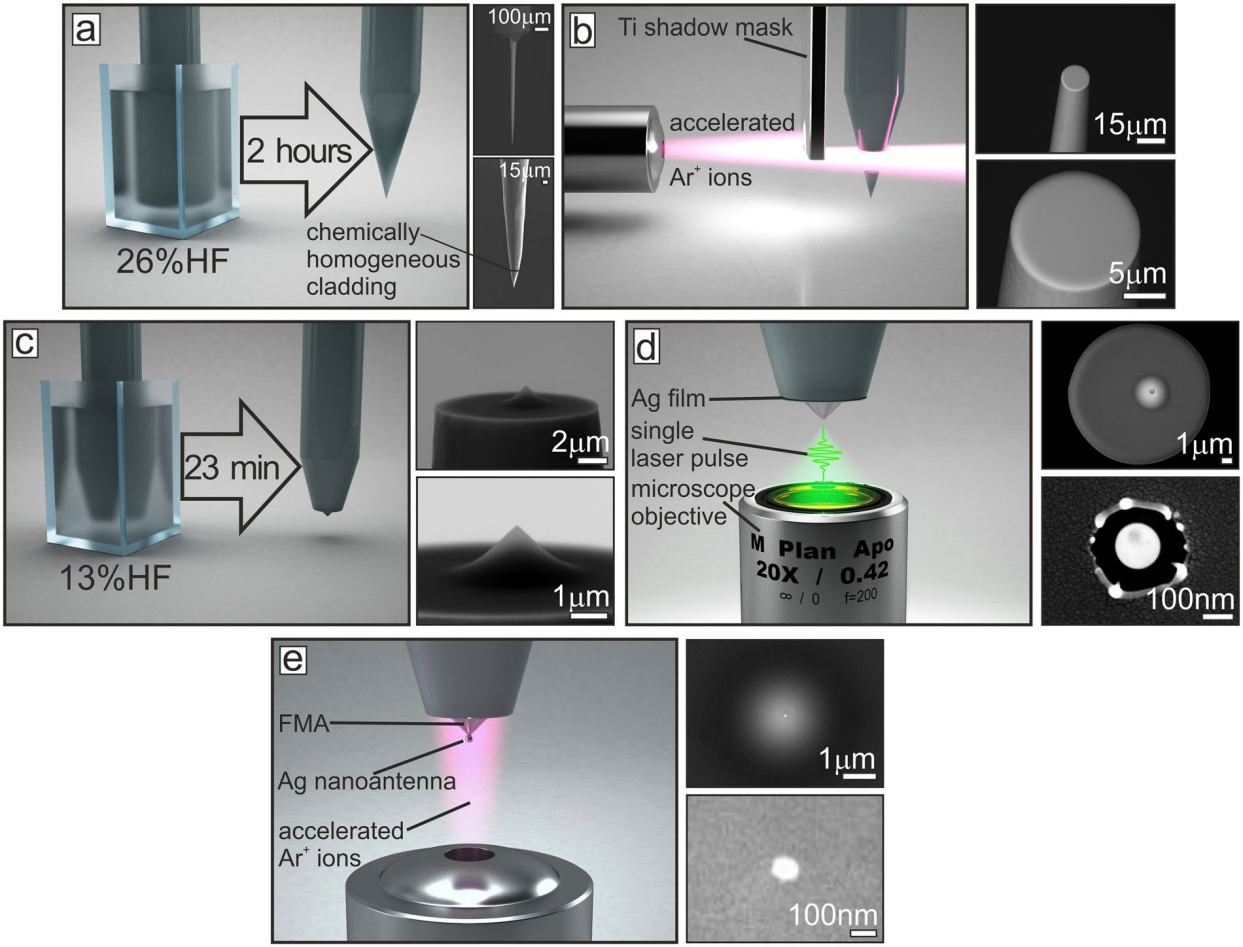
Fabrication of the scanning probe for spectrally-based RI mapping. Schematic illustration of the fabrication process of the RI mapping probe on the endface of the single-mode optical fiber including (**a**) chemical etching in the 26-% aqueous HF solution, (**b**) polishing with an accelerated Ar^+^-ion beam, (**c**) chemical etching in the 13-% aqueous HF solution, (**d**) ablation of the FMA apex with the ns-duration laser pulse and (**e**) final polishing with an accelerated Ar^+^-ion beam to remove non-ablated parts of the silver film from the FMA. Corresponding SEM images illustrating each step of the fabrication chain are given in the right part of each image.

The scanning probe is fabricated according to the following multi-step procedure. The FMA serving as rigorous support for the Ag NP nanoantenna and as a microlens collecting the scattering signal is obtained via a modified chemical etching procedure described elsewhere^22^. Briefly, the mechanically cleaved endface of the commercial optical fiber (OF, Thorlabs SM300) is first immersed into the aqueous HF solution (26% in water) for 2 hours. Such chemical etching produces the tapered fiber tip having full taper angle of ≈20° ^23^ and relatively rough surface (see Fig. 1(a)). Then, using an accelerated Ar^+^-ion beam (Hitachi IM4000), the tapered tip is cut off through a shadow titanium mask (Fig. 1(b) with insets I and II) at the point, where its diameter becomes smaller ≈20 *μ*m. Finally, the smoothed endface of the OF with the reduced diameter is etched in a weak 10-% aqueous solution of HF for 23 minutes, resulting in formation of the FMA with a full taper angle of ≈90° centered to the optical fiber core (Fig. 1(c) and insets I and II therein). According to our previous studies^22^, the corresponding geometry combines extremely high focusing capability (optical spot diameter of 370 nm) combined with the small focal distance (≈200 nm) ensuring efficient signal collection.

To produce the plasmonic nanoantenna, the FMA is coated with a 30-nm thick Ag film at a pressure of bar 5⋅10^−6^ and an average speed of 0.8 nm · s^−1^ using an e-beam evaporation procedure (Ferrotec EV M-6). Before deposition, the FMA surface was pre-cleaned with a build-in ion source (KRI EH200) to remove the residuals of etching process and provide better adhesion to the deposited material. Thereafter, the very tip of the FMA is irradiated with a single second-harmonic (532 nm) 7-ns FWHM pulse from a Nd:YAG laser system (Quantel Brio GRM Gaussian). Laser radiation spatially filtered by a segment of single-mode optical fiber (Thorlabs SM405) is focused onto the FMA tip by a microscope objective (Mitutoyo 20×) having numerical aperture of NA = 0.42 yielding in the diameter of the laser spot on the FMA tip of ≈1.22 *λ*(NA)^−1^ = 1.55 *μ*m (Fig. 1(d)). Before irradiating the FMA tip, the applied pulse energy E was precisely calibrated by ablating the surface of the similar 30-nm thick Ag film covering flat silica glass substrate. By taking into account the local near-field enhancement of the laser radiation near the Ag-coated tip, the pulse energy for tip ablation is chosen to be twice lower comparing to the case of the smooth Ag film of the same thickness (see Fig. S1 in the Supporting information). In this way, laser irradiation at pulse energy E = 4 nJ results in formation of the 350-nm wide through hole with the 150-nm diameter spherical-like nanoparticle formed from the transiently molten material (Insets I and II on the Fig. 1(d)). Finally, the remaining metal film covering non-irradiated parts of the FMA is polished away by the normally-incident Ar^+^ ion beam resulting in a separated Ag spherical-like nanoantenna with a slightly reduced diameter of 100 nm (Fig. 1(e) and insets therein). The parameters of the ion-beam polishing procedure are optimized according to out previous studies to avoid dewetting of the Ag film^24^. The geometric parameters of the probe on each step of the fabrication chain are controlled with a low-vacuum scanning electron microscopy (SEM, Hitachi S3400). The low-vacuum operation regime allows the surface charge to dissipate upon imaging of the dielectric probe eliminating the need for the conductive layer.

### Refractive index mapping

We integrate the fabricated FMA-based probe containing 100-nm diameter Ag NP on its tip into a standard tuning-fork feedback system of a home-build aperture-type SNOM^25^ to provide precise control over the distance between the nanoantenna and the sample surface. The FMA collects and directs the scattering signal from the Ag nanoantenna to a grating-type spectrometer (Andor, Shamrock 303i) equipped with a thermoelectrically cooled CCD-camera (Newton 971). The scanning is performed at constant height mode. Two experimental excitation schemes of the nanoantenna is realized in the present study. In the first one, the dark-field side illumination at an angle of 85° to the sample’s normal is performed to excite the DP mode of the Ag NP with a collimated p-polarized white-light radiation. The second scheme provides the nanoantenna excitation with a collimated p-polarized light at an incidence angle of 45° from the bottom side of the transparent sample. For both schemes, the radiation from a 300-W stabilized calibrated tungsten bulb used as a broadband white-light source is focused with a long-working-distance lens (MY100X–806 lens, NA = 0.7, working distance of 6 mm).

To produce the sample having several step-like RI jumps, the following procedure is performed. The 500-nm thick layers of the Al_2_O_3_ and MgF are consequentially coated onto the bulk 125-*μ*m thick SiO_2_ substrate using e-beam evaporation procedure, followed by the deposition of the 500-nm thick Cu protective layer with a magnetron sputtering. Then, the ion-beam polishing is performed to produce the cross-section cut of the layered sample. SEM and atomic-force microscopic (AFM) inspections are undertaken to access the surface roughness of the produced cut as well as check possible height variations near its layer boundaries. For subsurface mapping studies, the produced cross-section cut is over-coated with a 20-nm thick SiO_2_ film.

### FDTD Simulations

Scattering spectra from a single spherical-shape Ag NP and electromagnetic-field distributions near its surface are calculated using finite-difference time-domain simulations (FDTD, Lumerical Solutions package). The dielectric function of Ag is modeled using build-in software fitting of the experimental data from^26^. The constant NP diameter of 100 nm is considered in all calculation in accordance with the experiments made. For other dielectric materials (MgF, Al_2_O_3_, SiO_2_) used to simulate the step-like RI jump as well as subsurface mapping geometry, the constant values of the RI are used as follows n(MgF) = 1.34, n(Al_2_O_3_) = 1.75, n(SiO_2_) = 1.51^27^. A linearly-polarized total-field scattered-field source with the wavelength ranging from 330 to 650 nm excites the Ag nanoparticle under various incidence angles. The size of the square unit cell is as small as 0.5 × 0.5 × 0.5 nm^3^, while the computational volume is limited by perfectly matched layers. The FMA is not considered in the simulations, as its presence generally adds only a constant redshift to the spectral position of main NP’s resonances according to the previous studies^28^. This spectral shift is taken into account when comparison with the experimental results are made. Meanwhile, the size of the monitor collecting the scattering signal from the Ag NP is adjusted to fit the real numerical aperture of the FMA.

## Results

### FDTD analysis of the nanoantenna-substrate interaction

**Figure 2:**
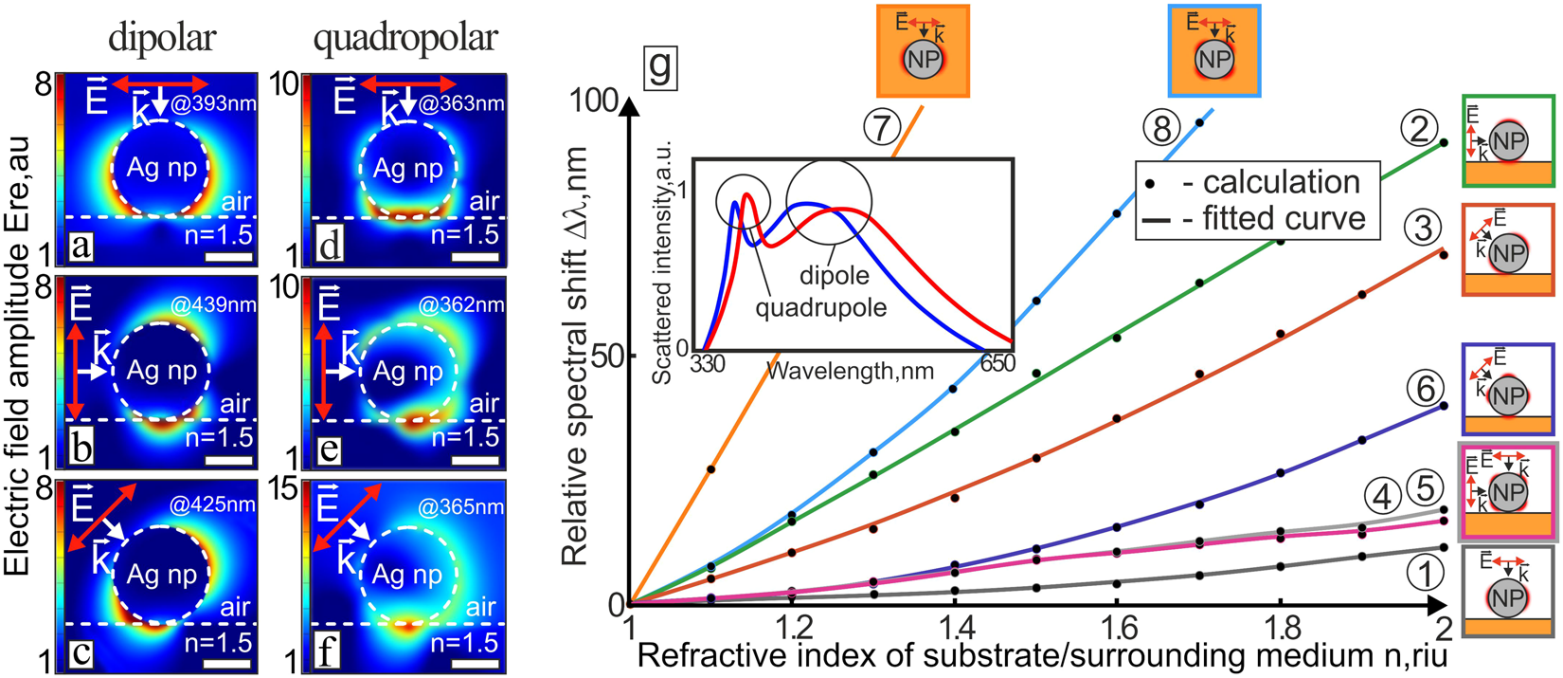
Numerical analysis of the nanoantenna-substrate interaction. Electric-field amplitude near the 100-nm diameter Ag NP placed above a semi-infinite dielectric substrate calculated under excitation of the DP (**a–c**) and QD (**d**–**f**) modes at various incidence angles of the p-polarized radiation. Scale bars correspond to 50 nm. Corresponding resonant wavelengths are indicated in each distribution. Red and white arrows indicate the polarization and wave vector directions, respectively. (**g**) Relative spectral shift of the DP and QD resonant wavelength versus the RI of the substrate (surrounding medium) calculated for a 100-nm diameter Ag NP under various excitation conditions. Curves 1–6 illustrate the cases shown on Fig. 1(a–f), respectively. Curves 7,8 were calculated for the DP and QD modes of the NP placed inside the homogeneous dielectric medium, respectively. Typical scattering spectra of the such Ag NP placed above the semi-infinite substrate with the RI n = 1.2 (blue curve) and n = 1.3 (red curve) are presented in the inset.

To start with, we have simulated the interaction of the Ag NP with an underlying substrate using FDTD calculations. Electric-field amplitude calculated for a “nanoparticle-substrate” system and related to the excitation of the DP and QD modes of the Ag NP under various irradiation directions reveals typical perturbation of the near-field distribution comparing to those for the nanoparticle in air (see Fig. 2(a–f) and Fig. S2 in the Supporting information). Such perturbations can be attributed to the interference of the reflected/scattered radiation with the incident light as well as to the common substrate effect^29^. Meanwhile, the characteristic features related to the near-field distributions for each resonance type can be still identified: two amplitude maxima arranged along the polarization direction in the case of DP resonance (Fig. 2(a–c)), and four maxima–in the case of QD one (Fig. 2(d–f)). As the relative orientation of the amplitude maxima near the NP is generally polarization-dependent for both types of resonances, their position with respect to the substrate can be tunned by providing the appropriate excitation conditions (polarization direction and incident angle) to increase the nanoparticle-substrate interaction, in its turn (see Fig. 2(b,f)).

Despite its simplicity, such formal representation correlates well with the relative spectral shift Δ*λ* of the DP (or QD) mode of the Ag NP calculated as a function of the RI (n) of the underlying dielectric substrate (see Fig. 2(g)) for various irradiation conditions similar to those previously presented in Fig. 2(a–f). Analysis of the presented data indicates that pronounced redshift of Δ*λ* = 91 nm/RIU (curve 2 in Fig. 2(g)) is observed for DP mode of the NP excited with the radiation polarized parallel to the surface normal. Similar tendency can be found also for QD resonance shift, when the angle between the polarization direction and substrate normal is equal to 45° (Fig. 2(f)). Under such conditions, an average slope of the Δ*λ*(n) dependence (curve 6 in Fig. 2(g)) reaches 40 nm/RIU providing the threshold sensitivity of 5 · 10^−4^ RIU to the corresponding variation of the substrate’s RI. To the contrary, overlapping of the characteristic amplitude maxima of the NP with the underlying substrate in the case of DP mode excited under normal irradiation (Fig. 2(a)), as well as for QD resonance - under its normal (or perpendicular) excitation (Fig. 2(d,e)), provides much weaker interaction between the nanoparticle and the underlying dielectric substrate. Such conditions, at which only the surface charges situated far from the substrate are excited and localized, provide relatively small spectral shift Δ*λ* with an average slope of 11 nm/RIU for a DP mode (curve 1, Fig. 2(g)) and ≈19 nm/RIU - for a QD one under both excitation directions (curves 4 and 5, Fig. 2(g)). It should be also stressed out that the characteristic amplitude maxima near the NP under its QD resonance excitation are more spatially localized owing to the smaller mode volume yielding in slightly weaker interaction with the underlying substrates. The different slopes of the relating Δ*λ*(n) dependences (curves 3 and 6, Fig. 2(g)) illustrate clearly this situation. Similar tendency remains even for the case of the same Ag NP fully surrounded by the homogeneous medium, with the averaged slopes of 311 nm/RIU and 152 nm/RIU for DP and QD localized surface plasmon resonances (LSPRs), respectively (curves 7 and 8 in Fig. 2(g)).

**Figure 3:**
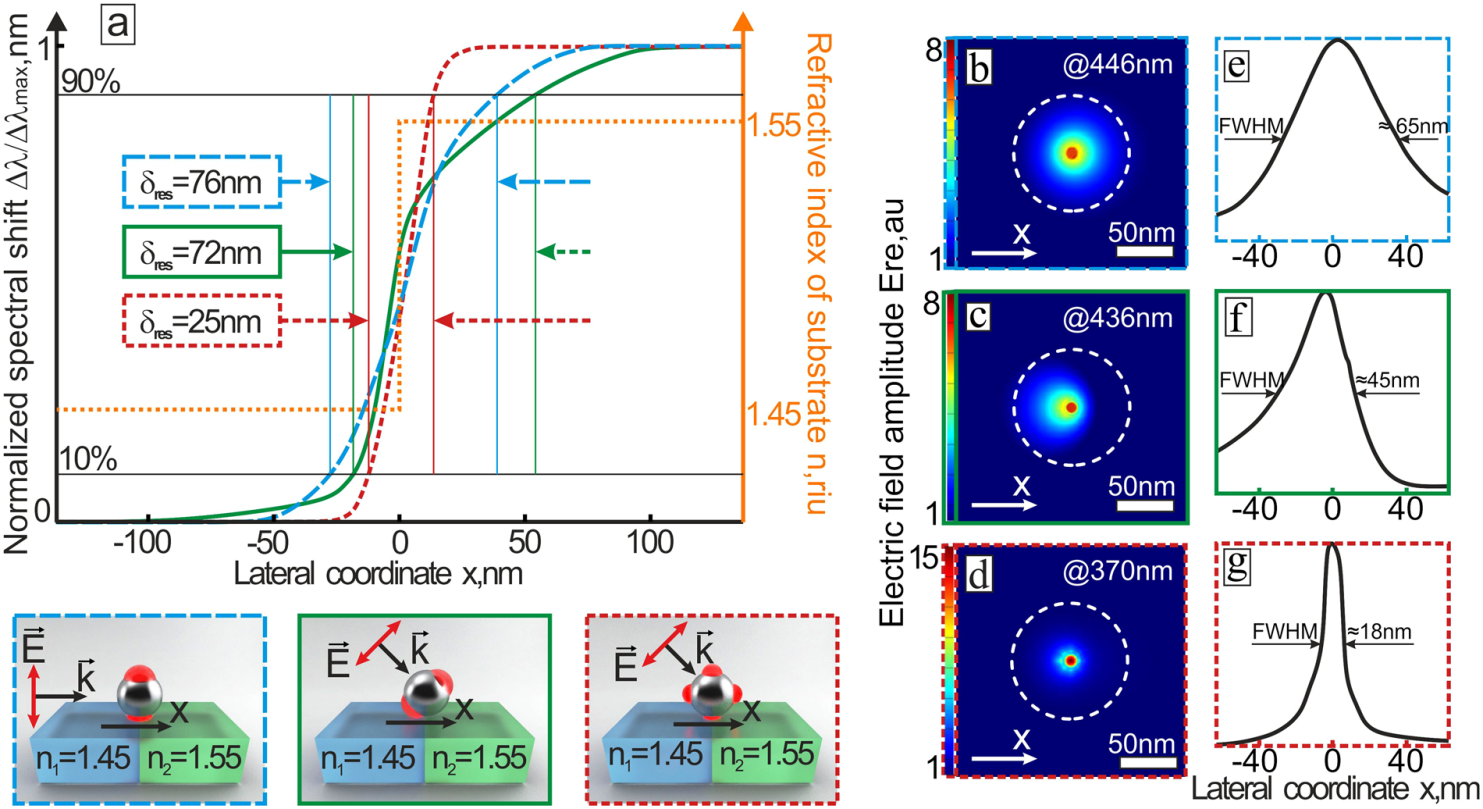
Lateral resolution of the spectrally-based RI mapping with the single plasmonic nanoantenna. (**a**) Normalized spectral shift of DP (blue and green curves) and QD (red curve) modes Δ*λ*/Δ*λ*_*max*_ as a function of the position along *x* axis. Orange curve shows the RI profile along the *x* axis, while the step-like jump of the RI corresponds to *x* = 0. The excitation scheme for each calculated case is presented on the inset images below. Corresponding electric-field amplitude (**b**–**d**) and central cross-section cuts along x axis (**e**–**g**) calculated 1-nm beneath the air-dielectric interface. Ag NP position above the dielectic substrate is marked with the white dashed circles. Scale bar corresponds to 50 nm.

Besides the QD mode demonstrates weaker response on the substrate’s RI variation, the stronger localization of the electromagnetic energy near the NP can be beneficial to achieve better lateral resolution. To illustrate this as well as to access the lateral resolution of the proposed RI mapping technique, we simulate the Ag NP, which moves at zero height along a linear direction (marked as x axis in the insets in Fig. 3(a)) on the dielectric surface crossing a step-like jump of the RI Δn = 0.1 (Δn = n_2_ − n_1_, n_1_ = 1.45, n_2_ = 1.55). Noteworthy, the lateral resolution *δ*_*res*_, which can be roughly estimated as a difference between the 10-% and 90%-levels of the maximal signal contrast, reaches 25 nm, when detecting the spectral shift of the QD LSPR of the Ag NP excited under side-irradiation at an angle of 45° (red dashed curve in Fig. 3(a)). In a sharp contrast, detection of the spectral shift of the DP mode excited at incidence angles of 0° or 45° (with respect to the sample’s normal), provides almost 3 times lower lateral resolution of ≈80 nm (blue and green dashed curves in Fig. 3(a)). Noteworthy, the similar calculations made for larger RI jumps (for example, Δn = 0.2 and 0.4) provide similar tendency and values of the lateral resolution for the DP and QD modes (see Fig. S3 in the Supporting information).

Interestingly, the estimated later resolution scales well with the characteristic E-field surface distribution calculated beneath the Ag NP (see Fig. 3(b–g)). Analysis of this calculated distributions also indicates an elongated asymmetric shape of the E-field “hot spot” beneath the Ag NP (Fig. 3(c,f)) in the case of the DP LSPR excitation under incidence angle of 45°, which appears to yield in the corresponding asymmetric behavior of the Δ*λ*/Δ*λ*_*max*_ dependence (green curve in Fig. 3(a)). Noteworthy, at fixed polarization direction, both resonances can be simultaneously tracked during the RI mapping, thus allowing combination of the high resolution with a high sensitivity. From this point of view, the detection of the spectral position of the DP LSPR excited under side-irradiation at an angle of 45° (green solid curve in Fig. 3(a)) provides the *δ*_*res*_ of 72 nm, while the RI sensitivity is almost twice higher that for the QD mode.

Finally, the electromagnetic field localized beneath a resonantly excited NP can penetrate to the underlying dielectric substrate to a certain distance, which potentially can be used to probe the subsurface features hidden by a nanometer-thin layer^30^. We have considered two most interesting cases by modeling the excitation of the DP mode at 0° incidence angle and QD one - at 45°, for the Ag NP underlying a two-layer substrate with the upper contact layer having variable thickness *d* (see Fig. S4 in the Supporting information). As expected, the lower mode volume of the QD mode results in almost twice lower penetration depth being compared to the DP mode according to the simulations made. Based on these results, we modeled then the system containing the two lateral step-like RI jumps (MgF, Al_2_O_3_, SiO_2_) hidden with a 20-nm thick SiO_2_ layer (see also Experimental section, for details). The simulations show the possibility to reconstruct the RI subsurface profile by detecting the spectral position of the DP mode at deep subwavelength resolution and agrees well with the experimental results presented further (see Experimental Results and Discussions).

### Experimental Results and Discussions

**Figure 4:**
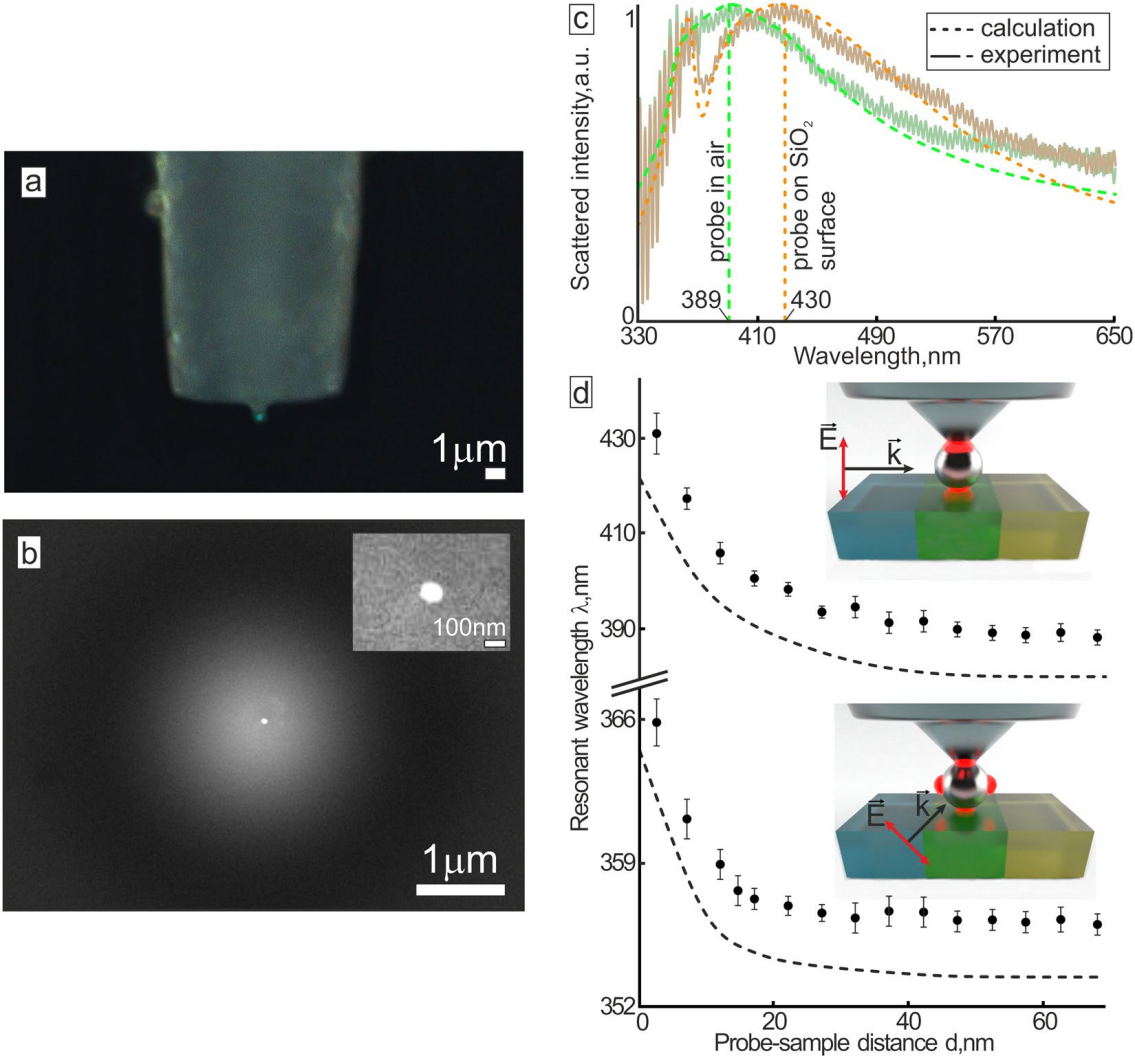
Scanning probe for RI mapping and its properties. Side-view optical (**a**) and SEM (**b**) images of the fabricated probe based on the FMA with the Ag NP attached to its tip. SEM image showing magnified view of the tip area with the 100-nm diameter Ag NP is given in the inset. (**c**) Experimental (solid curves) and calculated (dashed curves) normalized back-scattering spectra of the Ag NP corresponding to the case of the probe placed far from the sample surface (green curve) and attached to the SiO_2_ surface (brown curve). Both spectra are collected with the FMA coupled to the optical spectrometer. (**d**) Spectral position of the DP (upper curve) and QD (bottom curves) mode of the Ag NP as a function of the distance d between the probe and the sample surface (SiO_2_). Corresponding FDTD calculations of the similar dependencies (dashed curves) are given for comparison. Inset images show the corresponding excitation schemes.

The proposed RI mapping strategy is realized with the specially designed optical fiber probe having the single Ag nanoparticle atop (see Experimental section for fabrication details). The probe represents the high-quality FMA with a full taper angle of 90° (Fig. 4(a)) providing the rigorous support to the Ag NP during the scanning process as well as efficient collection of the scattered signal. The optical properties of the FMA are given elsewhere^22^. The 100-nm diameter Ag NP supporting the detectable DP and QD modes is produced on the very tip of the FMA using single-pulse laser ablation followed by the ion-beam polishing (Fig. 4(b)). Silver is chosen owing to its superior plasmonic properties, which provide the way to operate in the blue spectral region allowing better light localization and lateral resolution, in its turn. Indeed, the other plasmon-active materials as gold, copper, aluminum as well as their multicomponent alloys can be also used to fabricate similar nanoantenna^31–33^. The choice of the suitable nanoantenna material also is to be made by taking into account the probe storage time, as the silver nanostructures are known to degrade with time owing to oxidation^34,35^.

The DP mode excited with p-polarized white light at an incidence angle of 85° demonstrates detectable redshift (Δ*λ*_*max*_ ≈ 40 nm, Fig. 4(c,d)), when the Ag NP approaches the surface of the smooth SiO_2_ substrate. Similar observation can be found for QD LSPR (Fig. 4(d)) excited from the substrate side at an incidence angle of 45°, besides the resonant wavelength starts to react on the local change of the dielectric environment at a twice shorter distance, owing to the stronger mode localization approved by corresponding FDTD calculations (dashed curves in Fig. 4(c)).

**Figure 5:**
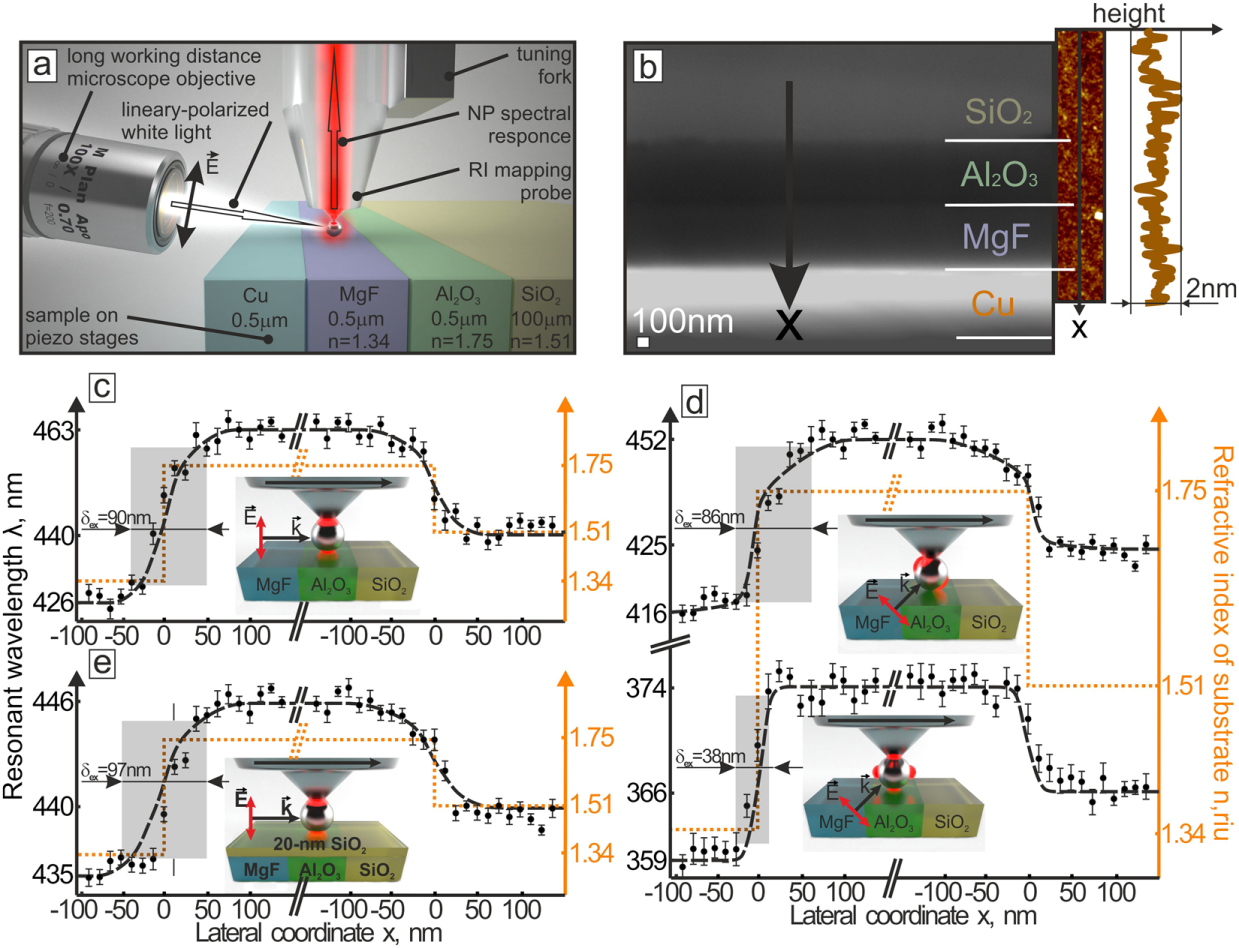
Benchmark experiment for lateral resolution measurements. (**a**) Schematic representation of the experimental setup used to access the lateral resolution of the RI mapping approach. (**b**) Normal-view SEM image of layered sample containing several jumps of the RI. The sample has relatively smooth surface according to the AFM analysis presented in the inset. The x axis indicates the scanning direction of the Ag NP along the sample surface. Scale bar of the AFM image is the same as for SEM one. (**c**,**d**) Spectral shift of the LSPR of the Ag NP as a function of the position along the scanning direction (*x* axis): shift of the DP mode excited under 85° with respect to the sample’s normal (**c**) and simultaneous shift of the DP and QD modes excited under 45° from the sample side (**d**). (**e**) Spectral shift of the DP LSPR of the Ag NP as a reaction on the two RI jumps hidden with 20-nm thick SiO_2_ layer. Dotted orange lines and blue dashed curves in (**c**–**e**) show the RI profile along the scanning direction and the results of comparative FDTD calculations, respectively. The excitation schemes for each presented case (**c**,**d**,**e**) are schematically illustrated on the inset images. Note slightly different scale along the y axis of the figures.

To proceed further with the properties of the fabricated probe, we performed a benchmark experiment by scanning the step-like RI jump allowing to access the lateral resolution (Fig. 5(a)). To do this, we fabricated the layered sample with two consecutive RI jumps (fabrication details are given in the Experimental section). The Ag NP is scanned across the cut of the layered sample having relatively smooth uniform surface (average height deviation ≈2 nm) including the areas near the RI jumps, as it was confirmed by comparative SEM and AFM studies (see Fig. 5(b)). The shear-force feedback maintains the Ag NP at a minimal height of about 1 nm during the scanning process to ensure strong coupling of the Ag NP to the dielectric substrate. Figure 5(c) shows the variation of the DP resonant wavelength of the Ag NP excited at an incidence angle of 85° (with respect to the sample’s normal) as a function of the lateral coordinate (x) along the scanning direction. Both RI jumps, which correspond to the local change of the dielectric environment (substrate material) beneath the Ag NP, can be recognized in the line scan via a corresponding spectral shift of the DP mode. The experimentally measured response agrees well with the corresponding FDTD calculation in terms of both the spectral shift value and the lateral resolution (≈90 nm) comparable with the size of the plasmonic nanoantenna (see Fig. 3(a) and dashed curve in Fig. 5(c)).

As it was mentioned, excitation of the Ag NP at an angle of 45° provides efficient coupling of the QD mode with the underlying sample surface, while the DP mode can also interact efficiently with the dielectric surface. Figure 5(d) demonstrates the variation of the resonant wavelength related to the DP and QD modes of the Ag NP excited with a p-polarized light as a function of the lateral coordinate *x* along the scanning direction. As seen, both modes react on the local change of the dielectric environment of the underlying surface, while remarkably better lateral resolution (≈40 nm) can be provided by mapping the position of the localized QD mode (see gray areas in Fig. 5(d)). Finally, to check the ability of the nanoantenna to feel the subsurface features, the RI jumps on the smooth sample surface are covered with a 20-nm thick SiO_2_ spacer and similar scanning procedure is performed by mapping the spectral position of the DP mode excited at an incidence angle of 85° (with respect to the sample’s normal). The detectable spectral shift of the DP mode was identified for both jumps showing the ability of Ag NP to reconstruct the subsurface RI profile. The value of the spectral shift on both RI jumps agrees well with the calculations (blue dashed curve in Fig. 5(e)) and is expectedly smaller being compared with the case when the spacer is absent. Meanwhile, lateral resolution of the mapping procedure remains almost unaffected by the presence of the spacing layer (see Fig. 5(c,e)).

The results presented consider the case of the simplest spherical-shape plasmonic nanoantenna, which interaction with the underlying dielectric substrate having relatively low RI is well understood. In particular, the substrate affects the electromagnetic field of the nanoantenna through its screening by the image charges induced inside the dielectric surface. The higher the RI of the substrate the stronger the potential generated by the image charges reduced by the factor of (*ε* − 1)/(*ε* + 1) where *ε* is the permittivity of the underlying dielectric medium^1,36,37^. From the first glance, higher RI jumps will induce strong redshift of both the DP and QD modes of the nanoantenna, which will be easier to identify in its scattering spectrum with the proposed approach. Meanwhile, high-RI substrate is expected to cause stronger modification of the characteristic spectrum of the nanoantenna owing to the plasmonic mode splitting^29^. Similarly, for the substrate made of plasmonic-active metals, even stronger polarization-dependent coupling between the nanoantenna and the underlying surface will occur resulting in the excitation of the surface-hybridized plasmon modes^38^. Such strong interaction was shown to dramatically perturb the characteristic spectrum of the spherical nanoantenna resulting in substantial spectral narrowing of its DP mode^38,39^. Taking into account the abovementioned effects, the spectral response of the plasmonic nanoantenna is expected to provide the way to resolve different types of materials as semiconductors and metals. Meanwhile, the corresponding analysis of the substrate-induced perturbations of the characteristic spectrum of the nanoantenna operating in a strong coupling regime appears to be more complex being compared to the case of the low-RI substrates and will become a subject of our ongoing studies.

Typically, the spectral linewidth of the corresponding type of plasmonic resonance is shape- and excitation-dependent. In this way, various complex designs of the plasmonic nanostructures^21,40^ can be considered to tailor their spectral linewidth and directivity allowing substantial improvement of the characteristics of the RI mapping approach. Additionally, resonant modes excited in the plasmonic nanoantennas typically have the broad linewidth owing to strong ohmic losses inherent to all plasmonic-active metals in the visible spectral range. In this way, one can also consider the low-loss all-dielectric nanoantenna as a promising candidate to replace the plasmonic one^41,42^. The all-dielectric resonators made of high-RI dielectric materials were recently shown to demonstrate strong substrate-induced perturbations of the scattering spectrum^43–45^, the effect which can be potentially used for spectrally-based RI mapping. Finally, combination of the low-Q plasmonic nanoantenna with a high-Q Fabry-Perot cavity providing discretization of the spectrally broad scattering signal by the narrow modes^46^ paves the way for further optimization of the mapping approach performance. Meanwhile, the fabrication of the micro-sized cavity coupled to the plasmonic nanoantenna within a single pointed nanoprobe allowing the scanning of an arbitrary sample surface can be technologically complex.

## Conclusions

Herein, we justify the approach to map the local RI of dielectric substrate at deep subwavelength resolution via detection of the surface-induced perturbation of the scattering spectra of a single plasmonic nanoantenna. The easy-to-implement lithography-free protocol allowing fabrication of the scanning probe containing the single spherical-shape Ag NP optically aligned to the fiber axicon microlens was suggested and approved. We attests the various excitation schemes of the plasmonic nanoantenna to provide efficient interaction of its DP and QD modes with the underlying sample surface. By taking advantages of the small mode volume of the QD mode and high sensitivity to the local dielectric environment of the DP one, it is possible to achieve superior lateral resolution in mapping of the local optical properties combined with the reasonably high sensitivity.

## Electronic supplementary material

Supplementary Information
