# Supplementary material for: Mapping the refractive index with single plasmonic nanoantenna
Source: Sci Rep. 2018 Mar 1;8:3861. doi: 10.1038/s41598-018-21395-w (PMC5832779; doi:10.1038/s41598-018-21395-w)
Supplement: Supplementary file 1 — Supplementary Information [file 41598_2018_21395_MOESM1_ESM.doc]

Supplementary Information

Mapping the refractive index with single plasmonic nanoantenna

S. Gurbatov^1,2,*^, O. Vitrik^1,2^, Yu. Kulchin^2^, A. Kuchmizhak^1,2^

^1^School of Natural Sciences, Far Eastern Federal University, Vladivostok, Russia

^2^Institute of Automation and Control Processes, Far Eastern Branch, Russian Academy of Science, Vladivostok

690041, Russia

[*gurbatov_slava@mail.ru](mailto:*gurbatov_slava@mail.ru)

**Note 1.** Here, we present the comparative E-field maps for dipolar and quadrupolar modes of the 100-nm diameter spherical Ag nanoparticle in air (upper row) and placed on the semi-infinite substrate having n=1.5 (bottom row) for the dipolar and quadrupolar modes. The maps show the substrate-induced distortion of the characteristic E-field distribution near the nanoparticle when it attaches the dielectric substrate.

***
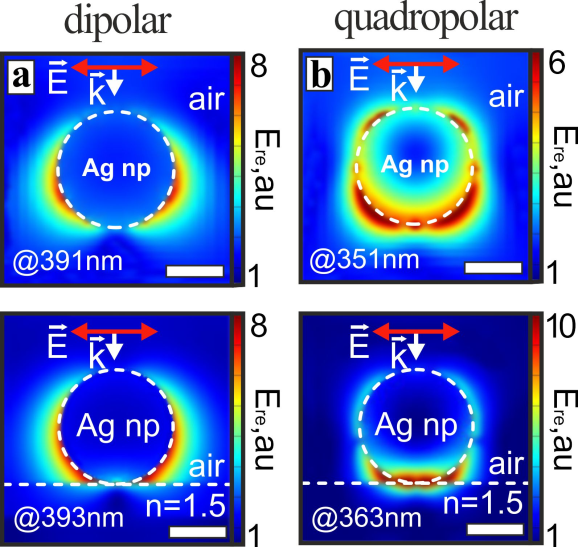
***

***Figure S1.*** *Electric-field amplitude E_re_ near the 100-nm diameter Ag nanoparticle in air (upper row) and on the semi-infinite dielectric substrate (bottom low) calculated under excitation of the dipolar and quadrupolar modes under p-polarized irradiation. Corresponding resonant wavelengths are indicated in each distribution. Red and white arrows indicate the polarization and the wave vector directions, respectively.*

**Note 2.** We calculated the enhancement of the electromagnetic field near the surface of the metal-coated glass fiber axicon having a full taper angle of 90^0^ under its irradiation with the laser pulse at λ=532 nm. Enhancement can be attributed to the mutual interference of the incident/reflected beam with the surface plasmon wave excited on the both sides of the 30-nm thick silver film covering the axicon surface. The 3-fold enhancement of the incident energy explains the lower melting/ablation threshold for the 30-nm thick Ag film on the axicon being compared to the case of the same film covering plane glass substrate.

*
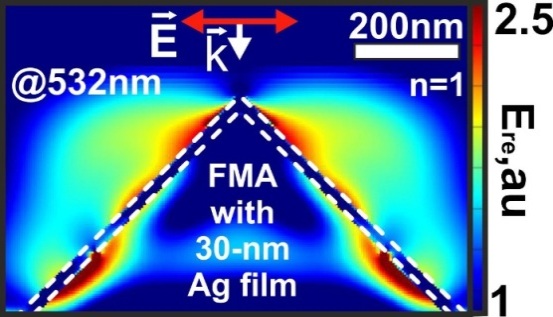
*

***Figure S2.*** *Electric-field amplitude near the fiber microaxicon tip covered with the 30-nm thick Ag film. The tip is irradiated normally with the p-polarized 1.55-μm diameter Gaussian beam at the wavelength of 532nm. Red arrow indicates the polarization direction.*

**Note 3.** Here, we present the normalized spectral position of the dipolar and quadrupolar modes Δλ/Δλ_max_ of the Ag nanoparticle moving, at zero height, along a linear direction (marked as x axis in Fig.S3(a,b)) on the dielectric surface and crossing the step-like jump of the refractive index Δn=0.2 (Fig.S3(a)) and 0.4 (Fig.S3(b)). For both presented cases, the shape of the dependence Δλ/Δλ_max_(n) reflecting the reaction of the Ag nanoantenna to the change of the local dielectric surrounding keeps the same shape and tendency, while the lateral resolution decreases slightly for higher RI jump of 0.4, owing to stronger reaction of the nanoantenna.

*
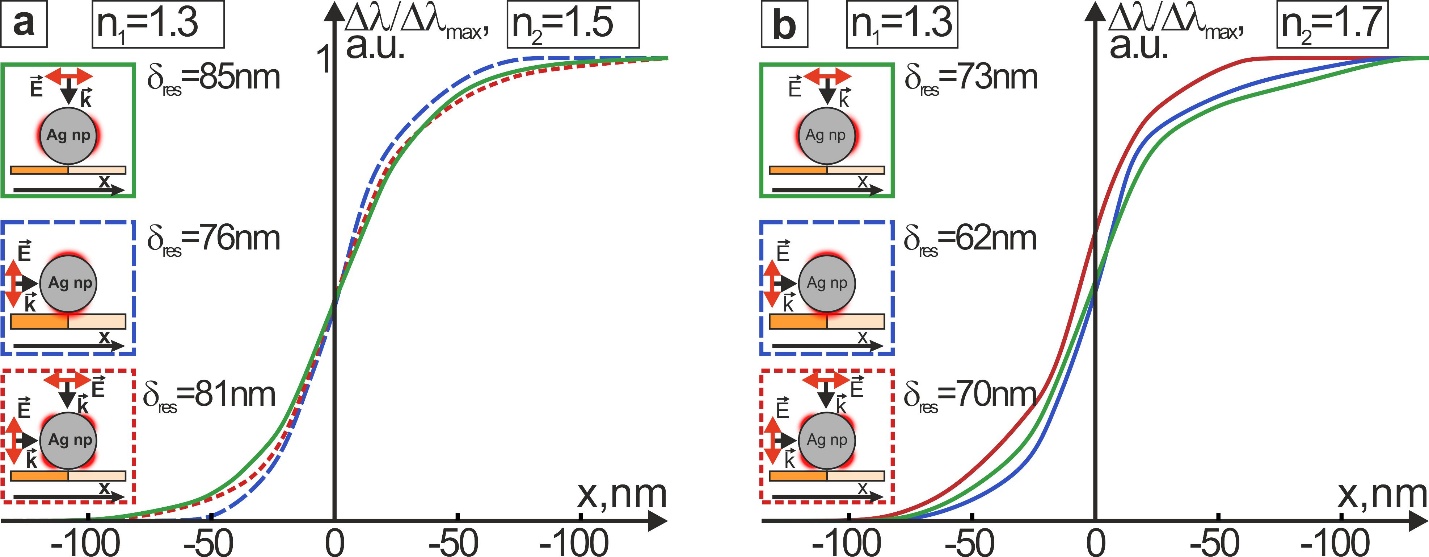
*

***Figure S3.*** *Normalized spectral shift of the dipolar (blue and green curves) and quadrupolar (red curves) modes as a function of the Ag nanoparticle position along lateral direction (x axis**). Step-like jump of the surface refractive index* *from 1.45 to 1.55 (a) and from 1.3 to 1.7 (b) of the substrate underlying the nanoparticle is at x=0. Corresponding insets show the excitation scheme for each calculated case.*

**Note 4.** To check the ability of the Ag nanoantenna to feel the hidden subsurface features, we have performed the simulation where we tracked the spectral position of the dipolar and quadrupolar modes of the nanoantenna lying above the double-layer dielectric substrate. In both cases, the optimal excitation schemes were used (see insets in the Fig.S4). The thickness of the top layer b is varied from 0 to 80 nm, while the refractive indexes of the top and bottom layers are swapped from 1.3 and 1.7. The simulations clearly show the twice longer distance at which the Ag nanoantenna feels the hidden subsurface layer for the case of the dipolar mode being compared to the quadrupolar one.

***
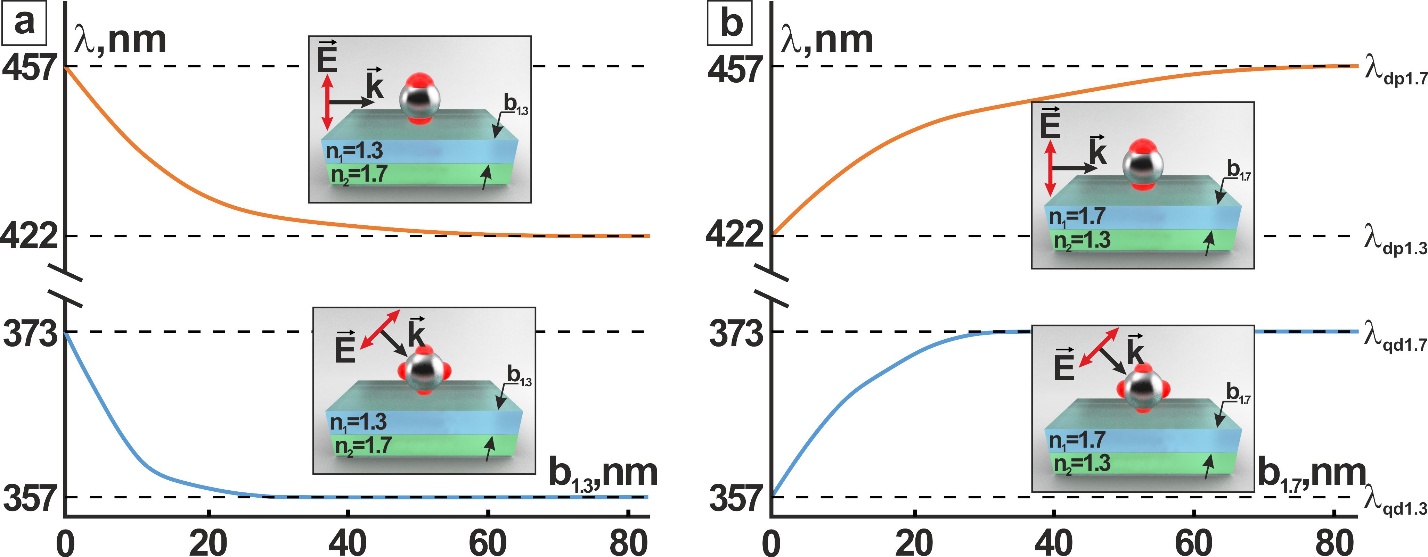
***

***Figure S4.*** *Spectral position of the dipolar (orange curves) and quadrupolar (blue curves) modes of the spherical Ag nanoparticle lying above the double-layer dielectric medium as a function of the top layer thickness b. The refractive indexes of the top and bottom layers are swapped between 1.3 and 1.7. Insets show* *the excitation schemes for each calculated case.*
